# A preoperative magnetic resonance imaging-based model to predict biochemical failure after radical prostatectomy

**DOI:** 10.1038/s41598-022-26920-6

**Published:** 2023-01-09

**Authors:** Minjie Pan, Shouchun Li, Fade Liu, Linghui Liang, Jinwei Shang, Wei Xia, Gong Cheng, Lixin Hua

**Affiliations:** 1 Department of Urology, The Affiliated Changzhou No. 2 People’s Hospital of Nanjing Medical University, Changzhou, 213011 Jiangsu Province China; 2 Department of Urology, The Affiliated Jiangning Hospital of Nanjing Medical University, Nanjing, 211100 Jiangsu Province China; 3 Department of Urology, First Affiliated Hospital of Nanjing Medical University, 300 Guangzhou Road, Nanjing, 210029 Jiangsu Province China

**Keywords:** Cancer, Diseases, Medical research, Risk factors, Urology

## Abstract

To investigate if a magnetic resonance imaging (MRI)-based model reduced postoperative biochemical failure (BF) incidence in patients with prostate cancer (PCa). From June 2018 to January 2020, we retrospectively analyzed 967 patients who underwent prostate bi-parametric MRI and radical prostatectomy (RP). After inclusion criteria were applied, 446 patients were randomized into research (n = 335) and validation cohorts (n = 111) at a 3:1 ratio. In addition to clinical variables, MRI models also included MRI parameters. The area under the curve (AUC) of receiver operating characteristic and decision curves were analyzed. The risk of postoperative BF, defined as persistently high or re-elevated prostate serum antigen (PSA) levels in patients with PCa with no clinical recurrence. In the research (age 69 [63–74] years) and validation cohorts (age 69 [64–74] years), the postoperative BF incidence was 22.39% and 27.02%, respectively. In the research cohort, the AUC of baseline and MRI models was 0.780 and 0.857, respectively, with a significant difference (*P* < 0.05). Validation cohort results were consistent (0.753 vs. 0.865, *P* < 0.05). At a 20% risk threshold, the false positive rate in the MRI model was lower when compared with the baseline model (31% [95% confidence interval (CI): 9–39%] vs. 44% [95% CI: 15–64%]), with the true positive rate only decreasing by a little (83% [95% CI: 63–94%] vs. 87% [95% CI: 75–100%]). 32 of 100 RPs can been performed, with no raise in quantity of patients with missed BF. We developed and verified a MRI-based model to predict BF incidence in patients after RP using preoperative clinical and MRI-related variables. This model could be used in clinical settings.

## Introduction

Globally, prostate cancer (PCa) is the second most frequent cancer and was the fifth leading cause of cancer death in men in 2020, with an estimated 1.4 million new cases and 375,000 deaths worldwide ^1^. Generally, radical prostatectomy (RP) is a valid treatment method for localized PCa ^2^. Elevated prostate serum antigen (PSA) levels are the most sensitive and specific early indicator of PCa recurrence after RP. Biochemical failure (BF) is defined as persistent, detectable PSA levels after RP (i.e., persistent PSA) or two consecutive PSA level increases of 0.2 ng/mL or more after a period of PSA normalization (i.e., biochemical recurrence). This scenario occurs in 30–40% of patients within 10 years after RP, and is associated with poorer cancer-specific outcomes ^3^.

Multi-parametric magnetic resonance imaging (mpMRI) is a highly sensitive tool for detecting clinically significant PCa ^4,5^. The approach also detects adverse pathological features in PCa patients, such as extracapsular invasion or lymph node metastasis ^6–10^. Some studies have reported that bi-parametric MRI (bpMRI), in contrast to mpMRI without dynamic enhancement, demonstrated a similar PCa diagnostic accuracy as mpMRI ^11^. Also, bpMRI is highly cost-effective when compared with mpMRI, and helps with diagnostic processes and risk stratification in PCa patients ^12^. In our study, we evaluated the added value of bpMRI for BF prediction in PCa patients. We developed and validated a pre-surgical model, which included bpMRI parameters and clinical variables, to predict BF.

## Methods

### Research and validation cohorts

**Figure 1:**
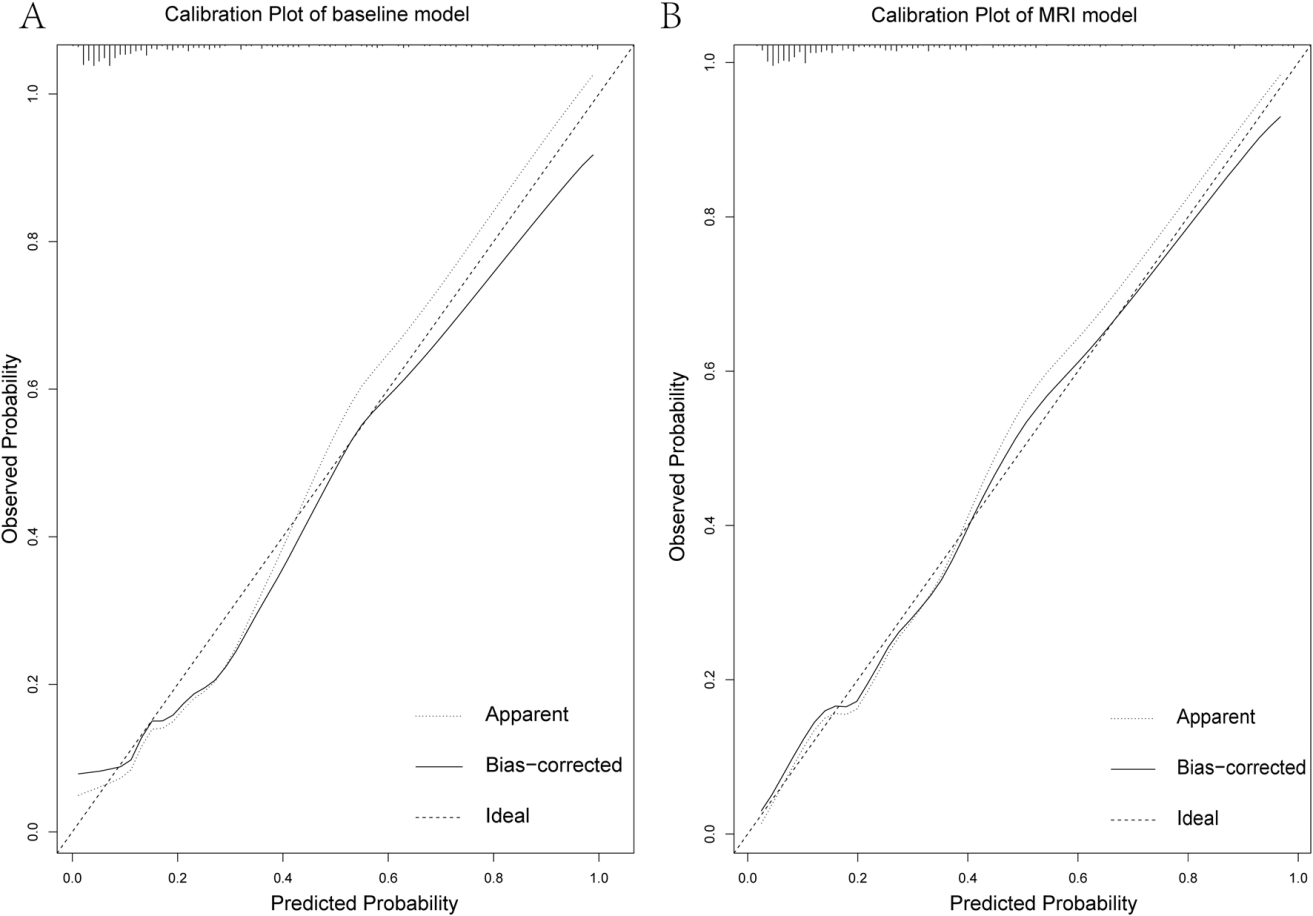
Calibration plot showing mean predicted risk in the validation cohort. (**A**) Calibration plot of the baseline model, (**B**) Calibration plot of the MRI model.

From June 2018 to January 2020, we retrospectively and consecutively analyzed 967 patients who underwent prostate bpMRI and RP. Exclusion criteria: (1) Patients who did not receive standardized MRI scans or underwent MRI scans at other centers; (2) Patients on neoadjuvant therapies; (3) Patients with transurethral resection of the prostate; (4) Patients who received hormone or radiotherapy after RP before BF; and (5) Patients with insufficient clinical data and undetermined PSA results from postoperative follow-up. Finally, 446 patients met our inclusion criteria (Fig. 1). Patients were then randomized into research (n = 335) and validation cohorts (n = 111) at a 3:1 ratio. All detected lesions were evaluated and classified according to PI-RADS v 2.1 guidelines ^13^. If a patient had multiple lesions in the same PI-RADS category, the lesion with the largest diameter was taken as an exponential lesion.

### BpMRI protocols

BpMRI was performed using a 3 T MRI system (Verio, Siemens, Germany), involving only T2WI and DWI (b value = 2000s/mm^2^), which were the dominant sequences used to characterize transitional and peripheral zones, respectively ^13^. The prostate volume was measured by bpMRI. All lesions were evaluated by senior personnel using PI-RADSv2.1 scores. The prostate MRI regional model was defined using the following four-zone method. To trisect the prostate along its axis, the lower third was defined as the apex zone while the upper third was the basal zone. The middle third was further divided into peripheral and non-peripheral zones. According to the four-zone method, a positive zone was defined as the major part of the lesion located or a lesion involved more than half of the zone. Therefore, patients with multiple lesions may also have multiple positive zones. Also, extracapsular extension (EPE) and seminal vesicle invasion (SVI) indices were recorded.

### Prediction model design

The baseline model embodies commonly used clinical variables comprising age at biopsy, body mass index (BMI), PSA at diagnosis, PSA density, suspicious digital rectal examination (DRE) (yes/no), biopsy pathology (ISUP grade), and surgical technique type (Robot-Assisted Radical Prostatectomy or Laparoscopic Radical Prostatectomy). The MRI model included these predictors, plus PI-RADS scores (1, 2, 3, 4, and 5), EPE at bpMRI (yes/no), SVI at bpMRI (yes/no), the zonal location of suspected lesions (apex region, basal region, central peripheral zone, and central non-peripheral zone), maximum diameter of the suspected lesion, and clinical stage (T1, T2, and/or T3). The outcome was BF. Postoperative PSA levels were initially measured at 1–2 months after RP, then at 3 month intervals in the second year, and intervals exceeding 6 months were deemed lost to follow-up.

### Statistical analysis

We developed and validated two multivariable logistic regression models to predict BF after RP. We recalibrated the risk model in the validation cohort by matching logistic regression with the logit of the predictive risk ^14^. A calibration slope near 1 indicated the correct predictive model fitting. The diagnostic correctness of both models was surveyed and balanced by the area under the curve (AUC) of the receiver operating characteristic (ROC). Model fitting was evaluated using calibration plots ^14^. False positive rates (FPR) and true positive rates (TPR) were used to evaluate the prediction accuracy of postoperative BF. The TPR indicated the ratio of patients with BF above the threshold, while FPR indicated the proportion of patients with non-BF values above the same threshold. The clinical value of the prediction model was weighed using the ratio of avoided BFs, the net benefit (NB), and a net reduction (NR) in false positives (FPs) ^15^.

We analyzed 95% confidence interval (CI) and SE values of prediction ability estimator in every predictive models, and the difference between the two models which from 2000 samples by stochastically selecting patients with substitution. We readjusted the prediction model and recalculated the prediction risk of every model in every sample in the research cohort. The 95% CIs came from 2.5% and 97.5% of the re-sampling distribution. Data for the resampling process included outcome (whether there was postoperative BF) and the unregulated predicted risk analyzed according to every risk models in the validation cohort. In every sample, the simple model for recalibration was readjusted, and then the predicted risk after calibration was recalculated. We compared variable distributions between research and validation cohorts. Categorical variables were assessed using χ^2^ tests, and we used Wilcoxon tests to analyze continuous variables. These tests were bilateral and a *P* < 0.05 value indicated statistical significance.

### Ethical approval and consent to participate

All methods were performed in accordance with relevant guidelines and regulations. This retrospective study received ethical approval from the Hospital Ethics Committee of the First Affiliated Hospital of Nanjing Medical University. Written informed consent was obtained from all subjects.

## Results

### Study population

In accordance with our exclusion criteria, we finally selected 446 consecutive patients. Then, we randomly divided 335 patients into the research cohort and 111 patients into the validation cohort, and both separately included in the model. Patient demographics in both cohorts are shown in Table 1. In research (median [inter-quartile range (IQR)] age = 69 [63–74] years) and validation cohorts (median [IQR] age = 69 [64–74] years), the postoperative BF incidence was 22.39% (n = 75) and 27.02% (n = 30), respectively. When compared with the validation cohort, age at biopsy, BMI, PSA, abnormal DRE, PI-RADS v2.1 category, ISUP grade, and surgical technique in the research cohort were similar.

**Table 1:** Patient Demographics of Research and Validation Cohort.

Variable	Research cohort (n = 335)	Validation cohort (n = 111)	*P* value^a^
Age at biopsy, Median (IQR)	69 (63–74)	69 (64–74)	0.495
BMI(Kg/m^2^), Median (IQR)	24.22 (22.49–26.03)	23.94 (22.15–26.67)	0.965
Preoperative PSA(ng/dL), Median (IQR)	10.53 (7.10–16.51)	10.13 (7.22–14.38)	0.579
PSAD(ng/ml^2^ ), Median (IQR)	0.30 (0.18–0.53)	0.27 (0.18–0.43)	0.275
Abnormal DRE, n (%)	65 (19.40)	16 (14.41)	0.237
**PI-RADS v2.1 category, n (%)**			
1	14 (4.18)	6 (5.41)	0.222
2	14 (4.18)	9 (8.11)	
3	89 (26.57)	20 (18.02)	
4	132 (39.40)	49 (44.14)	
5	86 (25.67)	27 (24.32)	
**ISUP grade, n (%)**			
GG1	101 (30.15)	31 (27.93)	0.685
GG2	69 (20.60)	20 (19.96)	
GG3	73 (21.79)	25 (21.97)	
GG4	77 (22.99)	32 (24.44)	
GG5	15 (4.48)	3 (4.04)	
**Surgical technique, n (%)**			
RARP	263 (78.51)	87 (78.38)	0.977
LRP	72 (21.49)	24 (21.62)	
**Postoperative status, n (%)**			
**Pathologic T category**			
T1	23 (8.06)	9 (12.61)	0.819
T2	260 (78.21)	83 (72.07)	
T3	52 (13.73)	19 (15.3)	
Margin status	119 (35.52)	41 (36.94)	0.788
Nodal status	7 (2.09)	4 (3.60)	0.392
Presence of intraductal carcinoma	3 (0.89)	1(0.90)	1.000
Biochemical failure n (%)	75 (22.39)	30 (27.02)	0.318

*DRE* Digital Rectal Examination; *IQR* Interquartile Range; *PI-RADSv2* Prostate Imaging-Reporting and Data System version 2; *PSA* prostate-specific antigen; *PSAD* PSA density; *ISUP* International Society of Urological Pathology; *GG* Grading Group; *RARP* Robot-Assisted Radical Prostatectomy ; *LRP* Laparoscopic Radical Prostatectomy; ^a^Comparison between research and combined validation cohorts.

The MRI characteristics for both cohorts are shown in Table 2. The research cohort had a similar zonal location of the index lesion, maximum diameter of the index lesion, MRI EPE, seminal invasion, and clinical stage when compared with the validation cohort (*P* > 0.05).

**Table 2:** MRI Characteristics of Research and Validation Cohort.

MRI variable	Research cohort	Validation cohort	*P* value
**Zonal location of index lesions**			
Negative MRI	28 (8.36)	15 (13.51)	0.111
Prostatic apex region	165 (49.25)	46 (41.44)	0.153
Basal region	42 (12.54)	15 (13.51)	0.789
Peripheral zone	171 (51.04)	54 (48.65)	0.662
Central non-peripheral zone	109 (32.54)	29 (26.13)	0.205
Maximum diameter of index lesion at bpMRI (cm), median (IQR)	1.10 (0.70–1.60)	1.10 (0.80–1.50)	0.275
MRI EPE, n (%)	43 (12.84)	14 (12.61)	0.951
Seminal invasion, n (%)	10 (2.99)	2 (1.98)	0.741
**Clinical stage, n (%)**			
T1	27 (8.06)	14 (12.61)	0.296
T2	262 (78.21)	80 (72.07)	
T3	46 (13.73)	17 (15.3)	

*EPE* Extraprostatic Extension.

### The prediction model

In the baseline model, PSA, GG3, GG4, and GG5 were independent predictors in terms of clinical variables, with statistical significance in the MRI model (**Table ****3**). The risk for BF was positively associated with PSA and increased with GG3, GG4, GG5, and lesion in the central peripheral zone. In research and validation cohorts, the calibration plot showed that the MRI model demonstrated a better fit when compared with the baseline model (Fig. 1).

**Table 3:** Logistic Regression Prediction Models of Biochemical Failure for Research Cohort.

Characteristic	Baseline model			MRI model		
	Coefficient	OR (95%CI)	*P* value	Coefficient	OR (95%CI)	*P* value
Intercept	1.106	3.023 (0.047–189.847)	0.599	1.905	6.722e + 00 (6.394e− 02—6.726e + 02)	0.417
Age at biopsy	− 0.036	0.965 (0.923–1.008)	0.111	− 0.035	9.568e− 01 (9.205e− 01—1.013e + 00)	0.150
BMI	− 0.093	0.911 (0.816–1.015)	0.095	− 0.111	8.952e− 01 (7.931e− 01—1.007e + 00)	0.068
PSA	0.048	1.050 (1.015–1.089)	0.007	0.047	1.048e + 00 (1.009e + 00—1.091e + 00)	0.017
PSAD	0.295	1.343 (0.413–4.159)	0.606	− 0.335	7.155e− 01 (1.953e− 01—2.519e + 00)	0.599
Abnormal DRE	0.482	1.619 (0.748–3.410)	0.211	0.132	1.141e + 00 (4.725e− 01—2.635e + 00)	0.762
**Surgical technique**						
RARP	NA	NA	NA	NA	NA	NA
LRP	0.261	1.298 (0.615–2.652)	0.482	0.201	1.223e + 01 (5.402e− 01—2.680e + 00)	0.620
**ISUP grade**						
GG1	NA	NA	NA	NA	NA	NA
GG2	0.521	1.683 (0.539–5.503)	0.371	0.302	1.352e + 00 (3.946e− 01—4.817e + 00)	0.631
GG3	1.182	3.260 (1.181 –9.999)	0.028	0.778	2.177e + 00 (6.707e− 01—7.681e + 00)	0.205
GG4	1.966	7.145 (2.791—20.969)	< 0.001	1.755	5.784e + 00 (1.990e + 00—1.937e + 01)	0.002
GG5	4.097	60.133 (11.908—476.667)	< 0.001	3.682	3.974e + 01 (6.481e + 00—3.557e + 02)	< 0.001
Prostatic apex region				0.568	1.764e + 00 (8.422e− 01—3.760e + 00)	0.135
Basal region				0.300	1.350e + 00 (4.589e− 01—3.745e + 00)	0.573
Central peripheral zone				0.912	2.490e + 00 (1.112e + 00—5.803e + 00)	0.030
Central non− peripheral zone				0.050	1.050e + 00 (4.116e− 01—2.615e + 00)	0.917
Maximum diameter of index lesion				0.474	1.607e + 00 (7.568e− 01—3.582e + 00)	0.229
MRI EPE				− 1.740	1.755e− 01 (3.243e− 03—6.841e + 00)	0.364
Seminal invasion				0.315	1.370e + 00 (1.225e− 01—2.730e + 01)	0.808
*PI-RADS v2.1 category*						
1				NA	NA	NA
2						
3				4.404	8.175e + 01 (1.475e− 72—NA)	0.875
4				4.878	1.313e + 02 (2.486e− 72—NA)	0.861
5				4.846	1.273e + 02 (4.078e− 72—NA)	0.862
*Clinical stage, n (%)*						
T1				NA	NA	NA
T2				− 6.311	1.816e− 03 (NA—9.966e + 70)	0.821
T3				− 4.011	1.812e− 02 (NA—1.827e + 72)	0.886

*DRE* Digital Rectal Examination; *IQR* Interquartile Range; *PI-RADSv2* Prostate Imaging-Reporting and Data System version 2; *PSA* prostate-specific antigen; *PSAD* PSA density; *ISUP* International Society of Urological Pathology; *GG* Grading Group; *RARP* Robot-Assisted Radical Prostatectomy ; *LRP* Laparoscopic Radical Prostatectomy.

**Figure 2:**
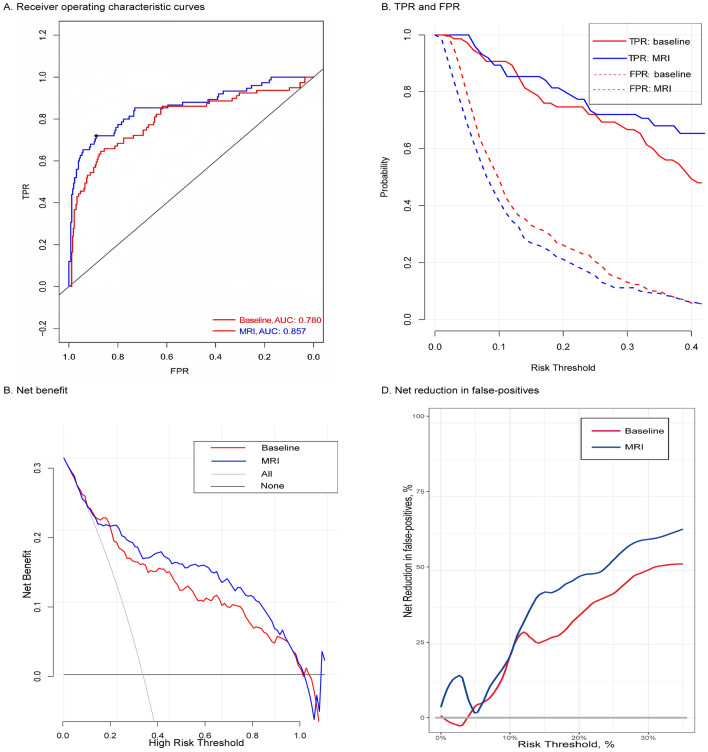
Plot showing the performance metrics of the research cohort. (**A**) Receiving operating characteristic curves or risk prediction models for CS prostate cancer, (**B**) TPR and FPR, (**C**) Net benefit (%), (**D**) Net reduction in false‐positives (%) of the three risk prediction models.

**Figure 3:**
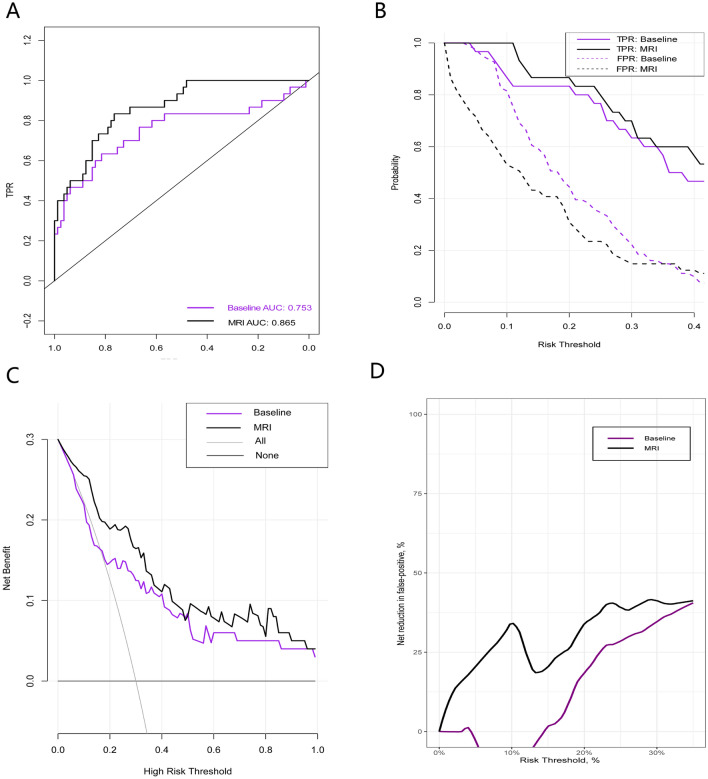
Plot showing the performance metrics of the validation cohort. (**A**) Receiving operating characteristic curves or risk prediction models for CS prostate cancer, (**B**) TPR and FPR, (**C**) Net benefit (%), (**D**) Net reduction in false‐positives (%) of the three risk prediction models.

When compared with the baseline model, the AUC increased from 0.780 to 0.857 (*P* < 0.05) in the MRI model in the research cohort (Fig. 2A and Table 4). In the validation cohort, when compared with the baseline model, the AUC increased from 0.753 to 0.865 (*P* < 0.05) (Fig. 3A and Table 5).

**Table 4:** Performance of the two Risk Prediction Models in the Research Cohort.

Performance parameter	Risk threshold, %	Model		Comparison
		Baseline	MRI	MRI vs baseline
AUC (95% Cl) TPR, % (95% Cl)	NA	78 (62–85)	86 (75–94)	8 (1 to 32)^a^
	10	99 (85–98)	85 (78–96)	-14 (-20 to 11)
	15	84 (78–98)	80 (72–96)	-4 (-9 to 1)
	20	86 (74–98)	81 (61–90)	-5 (-10 to 2)
FPR, % (95% Cl)	10	74 (28–94)	52 (14–60)	-22 (-39 to -8)
	15	58 (23–83)	41 (11–44)	-17 (-25 to 4)
	20	44 (14–62)	32 (10–40)	-12 (-29 to -1)
NB, % (95% Cl)	10	15 (11–24)	24 (13–34)	9 (-2 to 13)
	15	20 (13–32)	25 (15–36)	5 (-8 to 20)
	20	18 (9–30)	20 (12–32)	2 (0 to 5)
NRFP, % (95% Cl)	10	7 (0–12)	-1 (-4–8)	-8 (-14 to 3)
	15	13 (0–23)	8 (0–15)	-5 (-9 to 19)
	20	14 (2–30)	30 (0–50)	16 (8 to 26)
PABF, % (95% Cl)	10	60 (52–80)	80 (70–90)	20 (14 to 27)
	15	50 (41–82)	67 (61–93)	17 (9 to 27)
	20	57 (38–80)	69 (55–92)	12 (0 to 25)

*NA* not applicable; *NB* net benefit; *NRFP* net reduction in false-positives; *PABF* percentage of avoided biomedical failures; percentage of avoided biopsies; *TPR* true-positive.

rate. ^a^*P* < 0.05 for the comparison of AUCs.

**Table 5:** Performance of the two Risk Prediction Models in the Validation Cohort.

Performance parameter	Risk threshold, %	Model		Comparison
		Baseline	MRI	MRI versus baseline
AUC (95% Cl) TPR, % (95% Cl)	NA	75 (63–82)	86 (76–93)	11 (5 to 23)^a^
	10	100 (86–100)	87 (82–100)	− 13 (0 to 19)
	15	87 (80–100)	83 (72–97)	− 3 (0 to 28)
	20	87 (75–100)	83 (63–94)	− 3 (0 to 37)
FPR, % (95% Cl)	10	75 (29–96)	53 (16–63)	− 22 (− 67 to − 12)
	15	59 (22–82)	43 (13–47)	− 16 (− 69 to 0)
	20	44 (15–64)	31 (9–39)	− 13 (− 55 to 0)
NB, % (95% Cl)	10	17 (12–28)	23 (15–31)	6 (− 2 to 12)
	15	15 (10–25)	18 (12–29)	3 (0 to 10)
	20	14 (7–23)	18 (11–28)	3 (0 to 9)
NRFP, % (95% Cl)	10	13 (0–18)	0 (0–14)	− 13 (− 18 to 1)
	15	17 (0–28)	13 (0–20)	− 3 (− 10 to 2)
	20	19 (6–37)	32 (0–56)	13 (− 6 to 20)
PABF, % (95% Cl)	10	62 (56–85)	84 (71–94)	22 (8 to 38)
	15	55 (42–80)	66 (60–91)	11 (6 to 30)
	20	53 (33–76)	66 (53–90)	13 (0 to 31)

*AUC* area under the receiver operating characteristic curve; *FPR* false-positive rate; *MRI* magnetic resonance imaging; *NA* not applicable; *NB* net benefit; *NRFP* net reduction in false-positives; *PABF* percentage of avoided biomedical failures; *TPR* true-positive.

rate. ^a^*P* < 0.05 for the comparison of AUCs.

TPR and FPR values in models are shown in Fig. 2B for the research cohort. TPR and FPR values in calibrated risk models (Table 4) are shown in Table 5 and Fig. 3B for the validation cohort. The FPR of the MRI model was lower when compared with the baseline model, and the loss of TPR was the smallest.

### Decision curve analysis (DCA)

Figures 2C, D showed the NBs and NRs in the quantity of FPs for the research cohort, and Fig. 3C, D showed the NBs and NRs in the quantity of FPs for the validation cohort. We then applied the MRI model to the validation cohort. When compared with “treat all” and “treat none” methods (“all model” and “none model”), the NB of risk thresholds ≥ 15% was always higher for all models (Figs. 2C and 3C). For instance, at a 20% risk cut-off, the NB was 3 (95% CI: 0–9) in both models, 14 (95% CI: 7–23) in the baseline model, and 18 (95% CI: 11–28) in the MRI model, and the NR in the quantity of FPs was 0 in the “all model (treat all)”, 19 (95% CI: 6–37) in the baseline model, and 32 (95% CI: 0–56) in the MRI model. The NB of the MRI model was identical to 18 BFs/100 men with-out negative BFs, four more than the baseline model. When compared with BFs in all patients with positive MRI results, the NR in the quantity of FPs based on the MRI model was equivalent to 32 fewer false BFs/100 men, while the quantity of undiagnosed BFs did not increase. Overall, 66% (95% CI: 53%–90%) of “treat all” could be avoided, while 83% (95% CI: 63%–94%) of postoperative BFs were identified. In contrast, the baseline model avoided 53% (95% CI: 33%–76%) of "total treatment" at this threshold, and identified 87% (95% CI: 75%–100%) of postoperative BFs under this threshold.

## Discussion

With the emergence of different treatments for localized PCa, the preoperative risk stratification of PCa patients is extremely important. BF is an ideal early prognostic PCa predictor after RP. A previous study reported that BF occurred when tumor tissue residue at surgery (i.e., positive margin and/or subclinical lymphatic metastasis) or cancer had disseminated beyond the prostate and outside the surgical field at surgery (i.e., minimal residual disease) ^16,17^.

Several commonly used multivariate risk tools based on pre-diagnosed PSA, T stage by DRE, and biopsy grading group categories have been used to predict postoperative PSA results ^18,19^. Several studies reported that MRI-derived parameters in a risk model increased the accuracy of BCR prediction. For example, a multivariable model including MRI PIRADS, along with clinical and pathological variables, outperformed European Association of Urology classification and CAPRA scores for predicting BCR (C-index: 77% vs. 62% vs. 60%, respectively) ^20^. Moreover, in another study ^8^, a pre-surgical model incorporating PI-RADS, fusion-targeted biopsy grade, and extraprostatic extension on MRI showed better accuracy in predicting BCR (AUC = 0.68–0.71) when compared with the D’Amico classification (AUC = 0.66–0.71). However, these findings used BR as the endpoint, and persistent PSA levels (> 0.2 ng/ml) after RP also required preoperative intervention. In Soga et al*.,* three sub-groups were defined in terms of the D’Amico classification risk (low, intermediate, and high) and the GP score (Gleason score multiplied by PSA). No significant difference was observed in the non-BF rate between low risk and low GP score subgroups or intermediate risk and intermediate GP score subgroups. But the non-BCF rate of the high GP score subgroup was significantly lower when compared with the high-risk subgroup (42.1% vs. 66.1%, *P* = 0.008). Based on multivariate analyses, a high GP score (*P* = 0.001; Hazard ratio (HR): 3.78; 95% CI: 1.95–7.35) was a significant independent risk factor for BCF after prostatectomy. However, these prediction models were limited to clinical parameters ^21^. In previous studies, Teloken et al*.,* reported that transition zone location indicated a better BR-free survival after adjusting for poor clinicopathological features ^22^. Shin et al*.,* showed the zonal location of lesions by MRI, and in addition to the PI-RADS category, this was putatively helpful estimating postoperative BF risks ^9^. These studies confirmed the role of MRI in predicting BF, but they did not develop prediction models. When MRI parameters were included in our prediction model, we identified better model fitting and a higher diagnostic accuracy, avoided more BFs, and maintained a similar level of sensitivity to BFs in contrast with the baseline model.

We used DCA in both risk prediction models to compare the NBs of “treat none” with “treat all”. “Treat none” refers to RP for localized PCa, while “treat all” refers to neoadjuvant androgen deprivation, extended radical operation, and lymph node dissection. In clinical settings, the risk threshold of “treat all” may be determined after physicians and patients weigh and judge the relative hazards of aggressive treatment regimen and the benefits of determining postoperative BFs. So, there was no one risk threshold in deciding who demanded RP, but a series of risk thresholds. Because of higher adverse-effect profiles and the disputed curative effects of “treat all”, we selected high risk thresholds for our DCA. Our novel MRI model also demonstrated better calibration characteristics and higher NBs when compared with the baseline model. Our DCA data indicated that when index lesion locations on bpMRI were included in the prediction model, it showed better model fitting and a higher predictive accuracy, thereby decreasing unnecessary treatments while increasing BF sensitivity when compared with the baseline model.

## Study limitations

Our model data were similar to previous data. However, our study had several limitations; it was a retrospective, single center data study, and was internally validated. In addition, this study was based on bpMRI, which may have some bias compared with multi-parameter MRI. These factors may have caused some verification bias and the data may not be universally applied ^23^.

## Conclusions

Using preoperative clinical and MRI-related variables, we developed and verified a MRI-based prediction model which predicted BF incidence in patients after RP. This model could be helpful in clinical settings.

## Data Availability

All data generated or analyzed in this study are included in the published article and its supplementary information files.

## Abbreviations

DRE: Digital rectal examination
IQR: Interquartile range
PI-RADSv2: Prostate imaging-reporting and data system version 2
PSA: Prostate-specific antigen
PSAD: PSA density
ISUP: International society of urological pathology
GG: Grading group
RARP: Robot-assisted radical prostatectomy
LRP: Laparoscopic radical prostatectom
EPE: Extraprostatic extension
AUC: Area under the receiver operating characteristic curve
FPR: False-positive rate
MRI: Magnetic resonance imaging
NA: Not applicable
NB: Net benefit
NRFP: Net reduction in false-positives
PABF: Percentage of avoided biomedical failures; percentage of avoided biopsies
TPR: True-positive rate
